# Interdental Splints for Fractures of Inferior Maxilla

**Published:** 1870-04

**Authors:** Geo. L. Fitch

**Affiliations:** Dentist.


### ARTICLE VIII.

### *Interdental Splints for Fractures of Inferior Maxilla.*

By Dr. Geo. L. Fitch, Dentist.

Interdental splints in various forms have been used for many years, but owing to their complexity or to the difficulty that any one but a skilled mechanic would find in manufacturing or applying them, their use has been limited. Undoubtedly the best of these appliances has been the vulcanite splint used of late years, but the objection to this is, that none but a dentist could apply it, and but few dentists would be able or willing to take the responsibility of treatment in these cases.

Prof. F. H. Hamilton, M.D., many years ago proposed the use of gutta percha, a wedge shaped piece of this material being softened in warm water and placed between the molar teeth on each side, and then moulded around the crowns of these teeth with the fingers, while a bandage around the chin and over the head completed the dressing. The jaws being held apart, by the gutta percha, food could be introduced between the front teeth. Other surgeons have followed in his track with the use of gutta percha, but the jaw, with all the different plans, was held firmly in one position.

The advantage which vulcanite splints have had, is in allowing the patient the use of the jaw while the broken fragments are still held firmly in apposition ; their disadvantage, as stated above, the difficulty of applying them. I have recently succeeded in applying gutta percha to the same use as vulcanite, and a brief description will, I trust, put interdental splints into the hands of every man in the profession. Take a piece of dental gutta percha of length sufficient to reach around the dental arch as far back as the second molars on either side, and of width sufficient to reach one or two lines below the crowns of the teeth, resting on the gums when it shall have been moulded to its place. As this variety of gutta percha comes in thin sheets, two thicknesses may be used, a little heat and pressure with the fingers converts them into one. Now the broken fragments being held properly in place by an assistant, dip the gutta percha into water heated to a little below the boiling point, and while it is softened by the heat, mould it gently around the teeth and gums: as it hardens quickly, possibly it may have to be dipped the second time in the hot water before it can be nicely and smoothly adjusted. Allow it to remain in its place a moment or two, and then withdraw it and dip in cold water, and if there be any superfluous portions they may be clipped off with the knife or scissors. Next take two pieces of iron wire, a little less than ordinary telegraph wire in size, (and these should be previously prepared) and bend them into the shape of a horse shoe, or more like the letter V, with its angle cut off somewhat. Flatten out one end of this wire until it is about two thirds as wide as the splint where it goes over the molar teeth ; heat this flattened portion a little and lay it on the gutta percha; the flattened portion should extend as far as the end of the splint and as far forward as the angle of the mouth, through which it should protrude, and then bend backwards on a line with the outside of the cheek, and make it (the wire) as long on the outside of the mouth as on the inside. The wire being somewhat heated will readily press its way a little into the splint, and with a thin piece of gutta percha placed over it and smoothly plastered down, our design is completed. The wire outside of the mouth may be bent into different shapes so as to be more readily fastened to the piece of leather or pasteboard which goes under the chin. This latter piece in this, as in the vulcanite splint, being made to fit the under surface of the jaw, and securely fastened to the wire on either side. If I have succeeded in making my description plain, I think any surgeon could in this manner easily construct an interdental splint equal in every respect to vulcanite, and at an expense not to exceed twenty-five cents. The gutta percha exerts no deleterious influence in the mouth any more than vulcanite does, and it may be taken out and washed frequently to insure cleanliness. Dental gutta percha may be had at any dental depot, and of the majority of dentists throughout the land.—*Medical Gazette.*

